# Supplementary material for: Vitamin D Deficiency Does Not Affect Cognition and Neurogenesis in Adult C57Bl/6 Mice
Source: Nutrients. 2024 Sep 2;16(17):2938. doi: 10.3390/nu16172938 (PMC11396937; doi:10.3390/nu16172938)
Supplement: Supplementary file 1 [file nutrients-16-02938-s001.zip › Supplemental Data File S1.pdf]

Diet of 1 IU/g		
Ingredients	1 kg<br>Amount g/kg	3 kg<br>Amount g/kg
Casein, vitamin free	200	600
L-methionine	3	9
Corn Starch	529.4975	1588.4925
Sucrose	100	300
Oil	70	210
Cellulose	50	150
Mineral Mix	35	105
Vitamin Mix ViD deficient	10	30
Vitamin D3	0.0025	0.0075
Choline Bitartrate	2.5	7.5
Total	1000	3000

Diet of 0.1 IU/g		
Ingredients	1 kg<br>Amount g/kg	3 kg<br>Amount g/kg
Casein, vitamin free	200	600
L-methionine	3	9
Corn Starch	529.49975	1588.49925
Sucrose	100	300
Oil	70	210
Cellulose	50	150
Mineral Mix	35	105
Vitamin Mix ViD deficient	10	30
Vitamin D3	0.00025	0.00075
Choline Bitartrate	2.5	7.5
Total	1000	3000

Diet of 2.4 IU/g		
Ingredients	1 kg<br>Amount g/kg	10 kg<br>Amount g/kg
Casein, vitamin free	200	2000
L-methionine	3	30
Corn Starch	529.494	5294.94
Sucrose	100	1000
Oil	70	700
Cellulose	50	500
Mineral Mix	35	350
Vitamin Mix ViD deficient	10	100
Vitamin D3	0.006	0.06
Choline Bitartrate	2.5	25
Total	1000	10000

Supplemental Table S1. Diet Composition of the control (top), deficient (middle), and supplemented (bottom) groups.
