# Supplementary material for: Vitamin D Deficiency Does Not Affect Cognition and Neurogenesis in Adult C57Bl/6 Mice
Source: Nutrients. 2024 Sep 2;16(17):2938. doi: 10.3390/nu16172938 (PMC11396937; doi:10.3390/nu16172938)
Supplement: Supplementary file 1 [file nutrients-16-02938-s001.zip › Supplemental Data File S2.pdf]

1. Vitamin D (25(OH)D) serum measurement

The serum was analyzed via a Roche Cobas 8000 (e801) - an electrochemiluminescence binding assay measuring 3.0 - 120 ng/ml (or 7.5 – 300 nmol/L) to confirm vitamin D levels.

2. Blood 25(OH)D measurements confirmed deficiency

The average 25(OH)D serum level in the female VDDG (13.53 ng/mL ± 2.711) was significantly lower than the VDSG groups (55.08 ng/mL ± 10.53) (KS test; $p < .05$) and met criteria for deficiency according to the Endocrine Society and the European Food Safety Authority (EFSA) (<20 ng/mL or 50 nmol/L). There was no significant difference between the VDCG (34.27 ng/mL ± 4.343) and the VDDG or VDSG.

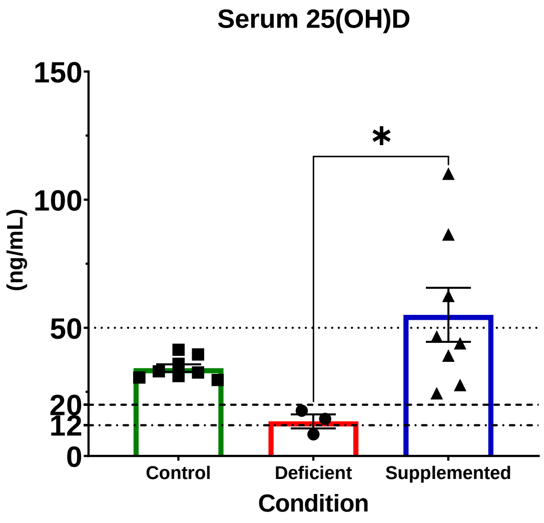

Supplemental Figure S1. Assessing Vitamin D blood serum levels in the different experimental groups. 25(OH)D (ng/mL) blood serum measurements in the female mice confirmed a significant decrease in the VDD group that reached deficiency levels. The supplemented group also had significantly more 25(OH)D than the deficient group. Dotted lines indicate cut-off values for deficiency by the Endocrine Society and the EFSA (<20 ng/mL), and the IOM (<12ng/mL), as well as insufficiency (IOM: between 12 and 20 ng/mL) and the "optimal" level (50 ng/mL) [92]. Data are expressed as mean ± SEM. *P*-values calculated by KS-test test are shown. * $p < 0.05$.
