# Supplementary material for: Vitamin D Deficiency Does Not Affect Cognition and Neurogenesis in Adult C57Bl/6 Mice
Source: Nutrients. 2024 Sep 2;16(17):2938. doi: 10.3390/nu16172938 (PMC11396937; doi:10.3390/nu16172938)
Supplement: Supplementary file 1 [file nutrients-16-02938-s001.zip › Supplemental Data File S3.pdf]

Steps for Iba1/GFAP Analysis including ImageJ script. Adapted from Young and Morrison [1]

For Both GFAP and Iba1:

1. Split Channels

2. Z-project: Max intensity (Project Max intensity the Z-slices that do not appear blurry)

3. ImageJ Macro: **Processing Part 1_**GFAPandIBA1.ijm:

```
run("Subtract Background...", "rolling=30");
run("Unsharp Mask...", "radius=3 mask=0.70");
run("Despeckle");
run("Despeckle");
run("Despeckle");
```

4. Adjust Brightness/Contrast (Improves ability to distinguish cells and background)

5. Manually Threshold to separate cells from background. Save as… in different folder.

6a. For Microglia: Processing Part 2 IBA1.ijm

```
run("Despeckle");
run("Close-");
run("Remove Outliers...", "radius=2 threshold=50 which=Bright");
run("Despeckle");
run("Close-");
run("Remove Outliers...", "radius=2 threshold=50 which=Bright");
```

6b. For Astrocytes: Processing Part 2 GFAP.ijm:

```
run("Despeckle");
run("Close-");
run("Remove Outliers...", "radius=2 threshold=50 which=Bright");
run("Despeckle");
run("Close-");
run("Remove Outliers...", "radius=2 threshold=50 which=Bright");
run("Despeckle");
```

```
run("Close-");
run("Remove Outliers...", "radius=2 threshold=50 which=Bright");
run("Despeckle");
run("Close-");
run("Remove Outliers...", "radius=2 threshold=50 which=Bright");
```

7a. Microglia Macro: Analyze Particles: Analyze Part_10um_Iba1.ijm:

```
// Set the directory path for your images and ROI files
dir = getDirectory("Choose a Directory ");
// Get the list of .tif files in the directory
list = getFileList(dir);
setBatchMode(true);
// Loop through each file
for (i = 0; i < list.length; i++) {
  if (endsWith(list[i], ".tif")) {
    // Open the .tif image
    open(dir + list[i]);
    // Derive the ROI file name from the image file name
    roiName = replace(list[i], ".tif", ".roi");
    // Check if corresponding ROI file exists
    if (File.exists(dir + roiName)) {
      // Open the ROI file
      roiManager("Open", dir + roiName);
      // Run the particle analysis
      run("Set Measurements...", "area mean standard modal min centroid center perimeter bounding fit shape feret's integrated median skewness kurtosis area_fraction stack display redirect=None decimal=3");
      run("Analyze Particles...", "size=10-Infinity display include summarize");
      // Save the results if needed
```

```
            saveAs("Measurements", dir + "Results_10px_Iba1" + list[i] + ".xls");
            // Close the image and ROI
            close();
            run("Close");
        }
    }
}
```

7b. Astrocyte Macro: Analyze Particles: Analyze Part_GFAP4um.ijm

```
// Set the directory path for your images and ROI files
dir = getDirectory("Choose a Directory ");
// Get the list of .tif files in the directory
list = getFileList(dir);
setBatchMode(true);
// Loop through each file
for (i = 0; i < list.length; i++) {
    if (endsWith(list[i], ".tif")) {
        // Open the .tif image
        open(dir + list[i]);
        // Derive the ROI file name from the image file name
        roiName = replace(list[i], ".tif", ".roi");
        // Check if corresponding ROI file exists
        if (File.exists(dir + roiName)) {
            // Open the ROI file
            roiManager("Open", dir + roiName);
            // Run the particle analysis
            run("Set Measurements...", "area mean standard modal min centroid center perimeter bounding fit shape feret's integrated median skewness kurtosis area_fraction stack display redirect=None decimal=3");
```

```
     run("Analyze Particles...", "size=4-Infinity display include summarize");

      // Save the results if needed

      saveAs("Measurements", dir + "Results_GFAP4px" + list[i] + ".xls");

      // Close the image and ROI

      close();

      run("Close");

    }

  }

}
```

8. Output will be given for each image in the folder. Lower limits for analyze particles was set at 4px for GFAP and 10px for Iba1 (3.1px/um at 20x magnification). Consolidate and compile .xls or .csv result of each subject in one folder.

A detailed explanation can of the analyze particles function in FIJI(ImageJ) can be found on the ImageJ website or at the following hyperlink: https://imagej.net/ij/docs/menus/analyze.html.

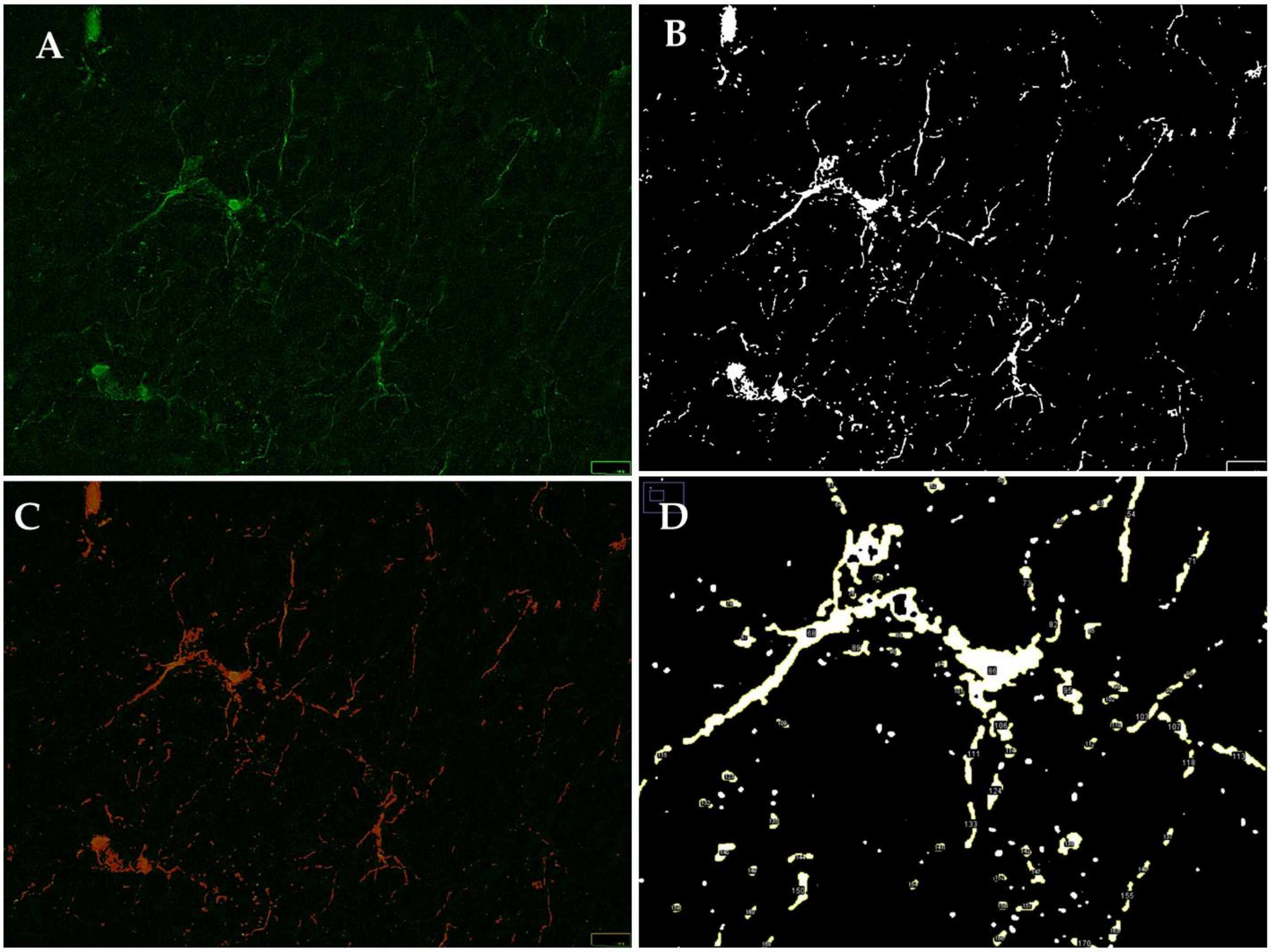

Supplemental Figure S2. Example of an image in the processing pipeline using a 50x-oil objective. (A) Iba1 (in green) in the DG of female C57BL6 mice after subtracting the background and sharpening the image. (B) The image is then thresholded to best separate the microglial (or astrocyte) cell body and processes from the background. After thresholding the image is despeckled and outliers with a small radius are removed. (C) The thresholded image overlay on the sharpened image of the microglial cells. (D) An example of the analyze particle function on FIJI; in this analysis microglial cells and processes under 10 pixels (~3um) were excluded from the analysis (based on trial-and-error thresholding) as they reflect background noise or very small processes that have been watershed by the thresholding process.

1. Young, K.; Morrison, H. Quantifying Microglia Morphology from Photomicrographs of Immunohistochemistry Prepared Tissue Using ImageJ. *J Vis Exp* **2018**, 10.3791/57648, doi:10.3791/57648.
